# 

**Published:** 1877-01

**Authors:** 


*Vol. XXXI.* **JANUARY, 1877.** *No. 1.*

THE

### DENTAL REGISTER,

A Monthly Journal Devoted
to the Interests of
the Profession.

EDITED BY J. TAFT, D. D. S.,

AND

PUBLISHED BY SPENCER, CROCKER & CO.,
OHIO DENTAL DEPOT,
No. 168 Race Street, Cincinnati, Ohio.

**TERMS: $2.50 Per Annum, in Advance; Single Copies 25c.**

J. P. GEPPERT, PRINTER.

